# White Light–Emitting Diodes (LEDs) at Domestic Lighting Levels and Retinal Injury in a Rat Model

**DOI:** 10.1289/ehp.1307294

**Published:** 2013-12-20

**Authors:** Yu-Man Shang, Gen-Shuh Wang, David Sliney, Chang-Hao Yang, Li-Ling Lee

**Affiliations:** 1 Institute of Environmental Health, National Taiwan University, Taipei, Taiwan; 2 Army Medical Department (retired), Fallston, Maryland, USA; 3 Department of Ophthalmology, National Taiwan University Hospital, Taipei, Taiwan; 4 Department of Ophthalmology, National Taiwan University School of Medicine, Taipei, Taiwan; 5 Green Energy and Environment Research Laboratories, Intelligent Energy-Saving Systems Division, Industrial Technology Research Institute, Hsinchu, Taiwan

## Abstract

Background: Light-emitting diodes (LEDs) deliver higher levels of blue light to the retina than do conventional domestic light sources. Chronic exposure to high-intensity light (2,000–10,000 lux) has previously been found to result in light-induced retinal injury, but chronic exposure to relatively low-intensity (750 lux) light has not been previously assessed with LEDs in a rodent model.

Objective: We examined LED-induced retinal neuronal cell damage in the Sprague-Dawley rat using functional, histological, and biochemical measurements.

Methods: We used blue LEDs (460 nm) and full-spectrum white LEDs, coupled with matching compact fluorescent lights, for exposures. Pathological examinations included electroretinogram, hematoxylin and eosin (H&E) staining, immunohistochemistry (IHC), and transmission electron microscopy (TEM). We also measured free radical production in the retina to determine the oxidative stress level.

Results: H&E staining and TEM revealed apoptosis and necrosis of photoreceptors, which indicated blue-light induced photochemical injury of the retina. Free radical production in the retina was increased in LED-exposed groups. IHC staining demonstrated that oxidative stress was associated with retinal injury. Although we found serious retinal light injury in LED groups, the compact fluorescent lamp (CFL) groups showed moderate to mild injury.

Conclusion: Our results raise questions about adverse effects on the retina from chronic exposure to LED light compared with other light sources that have less blue light. Thus, we suggest a precautionary approach with regard to the use of blue-rich “white” LEDs for general lighting.

Citation: Shang YM, Wang GS, Sliney D, Yang CH, Lee LL. 2014. White light–emitting diodes (LEDs) at domestic lighting levels and retinal injury in a rat model. Environ Health Perspect 122:269–276; http://dx.doi.org/10.1289/ehp.1307294

## Introduction

Artificial lighting is a basic element in modern society; however, the potential health risks caused by light pollution have increased with the development of more sophisticated lighting technology (Chepesiuk 2009). Among the wide variety of artificial lighting selections, light-emitting diodes (LEDs) emit higher levels of blue light than conventional light sources. These LEDs provide humans with their first exposure to such extensive blue light (Behar-Cohen et al. 2011). From an environmental health perspective, retinal light injury and the potential risks for chronic exposure from using LEDs as a domestic light source require assessment before further development of this important, energy-saving technology.

LED (or solid-state) lighting sources are designed to emit all energy within the wavelength range of human vision, making LEDs the most energy-efficient commercially manufactured light. However, many current “white-light” LED designs emit much more blue light than conventional lamps, which has a number of health implications, including disruption of circadian rhythms (Holzman 2010). The most popular LED lighting product, a phosphor-conversion (PC) LED, is an LED chip that emits blue light, which passes through a yellow phosphor-coating layer to generate the ultimate white light (Spivey 2011). Although the white light generated from LEDs appears normal to human vision, a strong peak of blue light ranging from 460 to 500 nm is also emitted within the white light spectrum; this blue light corresponds to a known spectrum for retinal hazards (Behar-Cohen et al. 2011). Some epidemiological studies have suggested that short-wavelength light exposure is a predisposing cause for age-related macular degeneration (AMD) (Wu et al. 2006). Animal models have also been used to determine that excessive exposure to blue light is a critical factor in photochemical retinal injury targeting photoreceptors and the retinal pigment epithelium (RPE) (Hafezi et al. 1997).

Photochemical retinal injury resulting from a cumulative effect is caused by free radicals generated from retinal tissue through continuous light exposure (Dong et al. 2006). When exposure surpasses the protective capability, unfavorable free radicals and reactive oxygen species may form (Wu et al. 2006). This enhances the oxygenated products and provides conditions favorable for photodynamic damage of photoreceptors and other retinal tissues (Beatty et al. 2000). However, the wavelength-dependent effect and its influences on white LED light-induced retinal degenerations remain unknown.

Retinal light injury was studied intensively after Noell et al. (1966) first described retinal damage caused by environmental exposure to fluorescent light, and numerous studies have reported that high-intensity blue light causes acute retinal injury (Ham et al. 1976). However, few studies have focused on retinal injury caused by exposure to relatively low-intensity blue light under chronic exposure conditions (Peng et al. 2012). The composition of the white-light spectrum differs among LED products, and their light qualities change over time. Although it is robust in the beginning, a PC LED progressively releases more short-wavelengths (blue light) when LED lumen depreciation occurs because of phosphor degradation. The quality of the light deteriorates after the lights pass the 70% lumen maintenance level (U.S. Department of Energy 2009). These characteristics suggest that a white LED can cause more blue light exposure than other domestic lighting sources. Cumulative exposure to blue light has been argued to accelerate aging of the retina and possibly play an etiological role in AMD (Behar-Cohen et al. 2011); thus, further study is needed to determine the potential retinal effects of domestic lighting with high blue light.

We hypothesized that chronic LED exposure may induce retinal photochemical injury. This study was performed in a rat model and the retinal neuronal cell damage caused by oxidative stress was examined. Functional, histological, and biochemical measurements were applied to identify the biomarkers for retinal light injury.

## Materials and Methods

**Figure 1:**
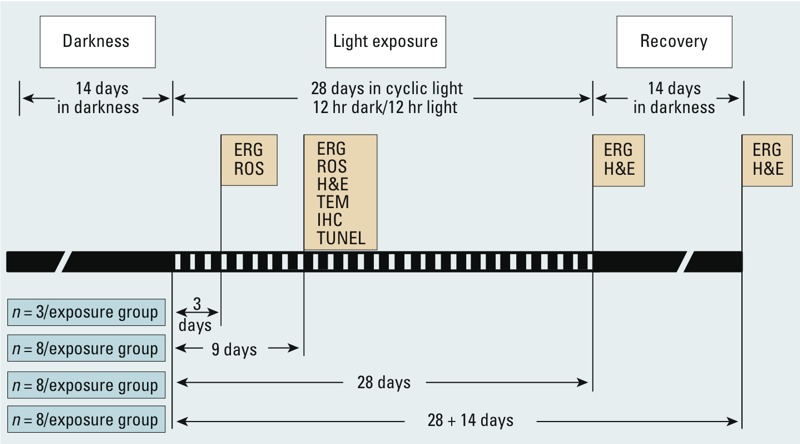
Timeline and experimental design. Abbreviations: CFL, compact fluorescent lamp; ERG, electroretinography; H&E, hematoxylin and eosin staining; IHC, immunohistochemistry; ROS, reactive oxygen species; TEM, transmission electron microscopy; TUNEL, terminal deoxynucleotidyl transferase dUTP nick end labeling. After 14 days of dark maintenance, the rats were divided into four groups and exposed to different light sources (blue LED, white LED, white CFL, or yellow CFL). Specific analytical techniques were performed at the end of exposure periods.

*Animals and rearing conditions*. We purchased a total of 120 adult (8-week-old) male Sprague-Dawley rats from BioLasco Taiwan Co. Ltd. (Taipei, Taiwan). Animals were housed in a dark environment for 14 days to clear the effect of light exposure from their previous rearing environment. Unexposed rats (remained in darkness) served as controls (*n* = 3 for each time point); the other 108 rats were separated into groups and received programmed light exposure from one of four light sources (*n* = 8 per exposure per time point) (Figure 1). All animals received food and water *ad libitum*. The use of rats in this study conformed to the *Statement for the Use of Animals in Ophthalmic and Vision Researc*h (ARVO 2013). The animals were treated humanely and with regard for alleviation of suffering. See Supplemental Material, p. 2 and Figure S1, for additional details.

**Figure 2:**
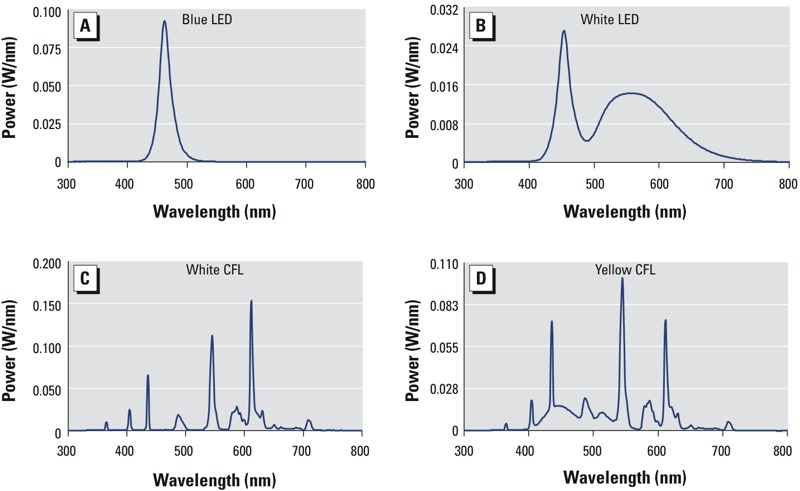
Light source SPD curves for (*A*) blue LED, (*B*) white LED, (*C*) white CFL, and (*D*) yellow CFL. The single-wavelength blue LED light (*A*) peaked at 460 nm (power of near 0.1 W/nm). White LED light (*B*) exhibited a CCT of 6,500 K. The first peak, which appeared at 460 nm with power of 0.028 W/nm, shows blue content; the bell shape of the second peak indicates higher yellow content. The SPD curve of white CFL light (*C*), with a CCT of 6,500 K, shows several sharp peaks across the spectrum; the blue peak is relatively shorter than the yellow or red peaks, and the full width at half maximum (FWHM) is smaller than that in (*A*) or (*B*). The SPD curve of yellow CFL light (*D*) is similar to that of white CFL (*C*), but with a CCT of 3,000 K; the highest peak represents yellow light. Although all of the light sources tested contain blue light peaks, the area under the curve variation leads to a difference in total intensity. Note the different scales for each light source.

*Light sources*. Single-wavelength blue LEDs (460 ± 10 nm) and PC white LEDs were custom made for the exposure experiments (BlueDog Technology Corporation Ltd., Taipei, Taiwan). The PC LED had a correlated color temperature (CCT) of 6,500 K. The CCT of the white compact fluorescent lamps (CFLs) (ESE27D-EX; Chuan Shih Industrial Corporation Ltd., Chuang-Hua, Taiwan) was also 6,500 K; whereas the CCT of the yellow CFLs (ESE27L-EX; Chuan Shih Industrial Corporation Ltd.) was 3,000 K. Each light source was programed for 40 measurements in an integrating sphere. The spectrum power distributions (SPDs) and total intensities for all light sources were tested by the Industrial Technology Research Institute (Hsinchu, Taiwan), a Certification Body Testing Laboratory, and are shown in Figure 2.

*Light exposure*. For light exposure, the animals were divided into four groups. Each rat was housed in an individual transparent cage (45 cm × 25 cm × 20 cm), and each cage was placed in the center of a rack shelf (75 cm × 45 cm × 35 cm). The light sources were set on the top of each shelf and were measured 20 cm away from each source to acquire the common domestic luminance level of 750 lux. After 14 days of dark maintenance, the light exposure started at 1800 hours on day 15, with total exposure duration of 3, 9, or 28 days under 12-hr dark/12-hr light cyclic routines. The animals were sacrificed at the end of light exposure, except for 32 animals (8 from each exposure group) that were exposed to light for 28 days and then returned to a dark environment for 14 days of recovery (28+14 group) to allow for possible removal of necrotic photoreceptor cell debris.

*Electroretinography (ERG)*. ERG was performed as described previously by Schatz et al. (2012) with some modification. Briefly, retinal electrical responses were recorded immediately before exposure began and after light exposure (allowing 18-hr dark adaptation for each rat before each ERG measurement) using ERG (RETIport ERG/VEP and RETIport software, version 4.7.2.8; Acrivet, Hennigsdorf, Germany). Alcaine (0.5%) (proxymetacaine hydrochloride; Alcon Pharmaceuticals Ltd., Puurs, Belgium) was applied for local anesthesia. Each 20-msec flash was provided by a 4 W LED (1 mV), and the illumination was set at 2.5 log cd^.^sec/m^2^ (candela-seconds per meter squared) for Scotopic ERG response. The final detection values presented are the weighted average of 10 stimulations as computed by the software program. See Supplemental Material, pp. 4–5, for additional details.

*Tissue collection.* Immediately after the ERG scans, animals were sacrificed with pentobarbital sodium (> 60 mg/kg, intraperitoneal) and eyes were enucleated. For hematoxylin and eosin (H&E) staining and terminal deoxynucleotidyl transferase dUTP nick end labeling (TUNEL), eyes were immersion-fixed overnight in 4% paraformaldehyde in 0.1 M phosphate-buffered saline (PBS), pH 7.4, and then embedded in paraffin. For immunohistochemical (IHC) staining, eyes were frozen immediately in liquid nitrogen; 4-μm cryosections were placed on glass slides and maintained at –80°C until analysis. For the reactive oxygen species (ROS) assay, enucleated eyes were frozen immediately in liquid nitrogen; each eye was homogenized in 500 μL saline for extraction. For transmission electron microscopy (TEM) analysis, eyeballs were immersion-fixed in 2.5% glutaraldehyde in PBS for 2 hr before processing.

*Hematoxylin and eosin (H&E) staining*. Briefly, tissues embedded in paraffin were cut in 5-μm sections and placed on glass slides; after deparaffinization, tissues were stained with H&E. Retinal histology was performed for the 9-, 28-, and 28+14-day light-exposure groups as described previously by Collier et al. (2011), with some modifications. We examined the midsuperior aspect of the retina for all histological analyses. We quantifid the outer nuclear layer (ONL) and examined alterations in retina morphology using a light microscope.

*TUNEL assay*. To detect apoptotic cells in eyes after 9 days of light exposure, the TUNEL assay was performed using a FragEL™ DNA fragmentation detection kit (Calbiochem, Darmstadt, Germany) following the manufacturer’s protocol for paraffin sections, with some modifications. Tissues were counterstained with DAPI (4´,6-diamidino-2-phenylindole). We used FITC (fluorescein isothiocyanate)-avidin D to label DNA strand breaks. Sections (the entire retina excluding the RPE layer) were visualized on a fluorescent microscope (Nikon Instruments Inc., Melville, NY, USA). The number of TUNEL-positive cells for each section was counted by Image-Pro Plus software (version 6.0; Media Cybernetics Inc., Rockville, MD, USA). See Supplemental Material, pp. 5–6, for additional details.

*IHC*. IHC was performed on eye tissue from the 9-day light-exposure group, as described previously (Collier et al. 2011; Fang et al. 2013). Briefly, cryosections of the retina samples were incubated overnight at 48°C with one of three primary antibodies: anti-8-hydroxy-2´-deoxyguanosine [8-OHdG; 1:50; JaICA (Japan Institute for the Control of Aging), Shizuoka, Japan] to detect DNA; anti-acrolein (1:200; Advanced Targeting Systems, San Diego, CA, USA) to detect lipids; and anti-nitrotyrosine (1:200; Abcam, Cambridge, MA, USA) to detect proteins. We used biotinylated anti-rabbit IgG as the secondary antibody, and FITC-avidin D to amplify the signal. The number of positive cells in each section was counted using Image-Pro Plus software.

*Transmission electron microscopy (TEM) analysis*. TEM of retinal tissues from the 9-day light-exposure group was performed at the Electron Microscopy Facility at the Department of Pathology at National Taiwan University Hospital, as described previously (Hafezi et al. 1997). Briefly, 1-mm retina slices were processed for TEM (for details, see Supplemental Material, pp. 6–7), and sections were examined using a high-resolution TEM instrument (JEM-1400; JEOL, Tokyo, Japan) at 80 kV.

*Free radical assay (ROS).* ROS were measured after 3 or 9 days of light exposure, as described previously (Fang et al. 2013). Briefly, ROS in retinas were quantified in the 3-day and 9-day light-exposure groups after adding the enhancer, lucigenin (bis-*N*-methylacridiniumnitrate), to the chemiluminescence analyzer (CLA-FS1; Tohoku, Tokyo, Japan). The stimulated superoxide anion (O_2_^•–^) and total oxidative products were captured every 10 sec and computed for 7 min after 1 min of baseline detection. See Supplemental Material, p. 7, for additional details.

*Statistical analysis*. Data are presented as the mean ± SD unless otherwise stated. Data were evaluated using analysis of variance (ANOVA), with Tukey post hoc tests to show differences between the groups. A *p*-value < 0.05 was considered to be statistically significant.

## Results

**Figure 3:**
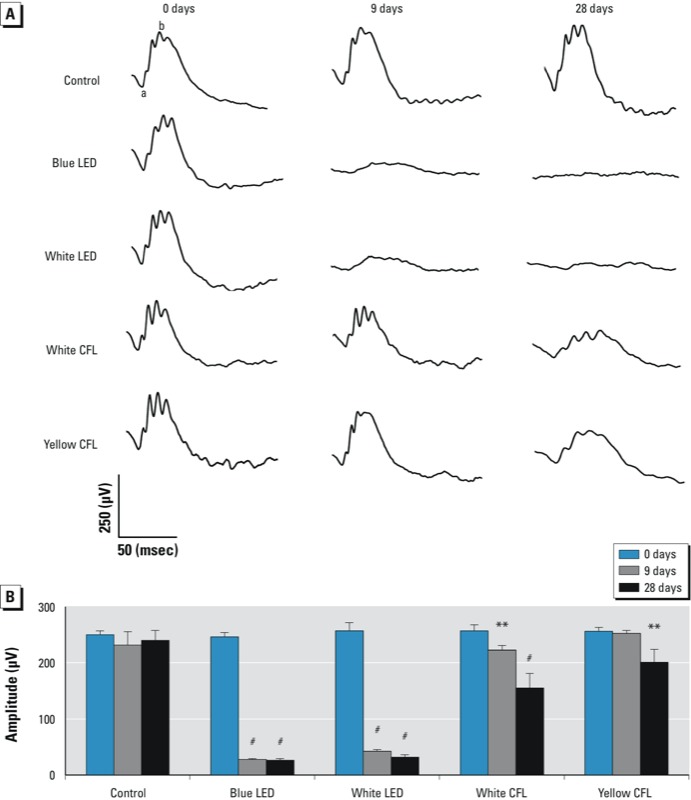
Representative ERG responses (*A*) and ERG b‑wave amplitude (*B*) in unexposed (control) rats or rats exposed to blue LED, white LED, white CFL, or yellow CFL at 0 (baseline), 9, or 28 days of light exposure. Values shown in (*B*) are mean ± SD (for each time point, *n* = 3 controls and 8 for each light-exposure group at each time point. **p < 0.01, and ^#^*p* < 0.001, compared with the control group by ANOVA and Tukey post hoc test.

*Electrophysiological response (ERG).* Representative ERG response curves of rats are shown in Figure 3A. After 14, 23, or 42 days of dark maintenance, the control retina showed a high b-wave peak, but the retinas from LED- and CFL-exposed animals had a low b-wave peak, indicating cell function loss. As shown in Figure 3B, the two LED groups and the white CFL group all demonstrated a significant decrease of b-wave amplitude at days 9 and 28 of light exposure (*p* < 0.001, by ANOVA followed by Tukey post hoc test). The b-wave amplitude of the yellow CFL group was not significantly decreased at day 9; however, it had decreased 21% at day 28. The data from each of the four exposure groups was not statistically different at 28+14 days compared with 28 days of exposure; this trend was also present in the H&E staining results (data not shown). Because we found no significant development after 3 days of light exposure, these data are not shown.

**Figure 4:**
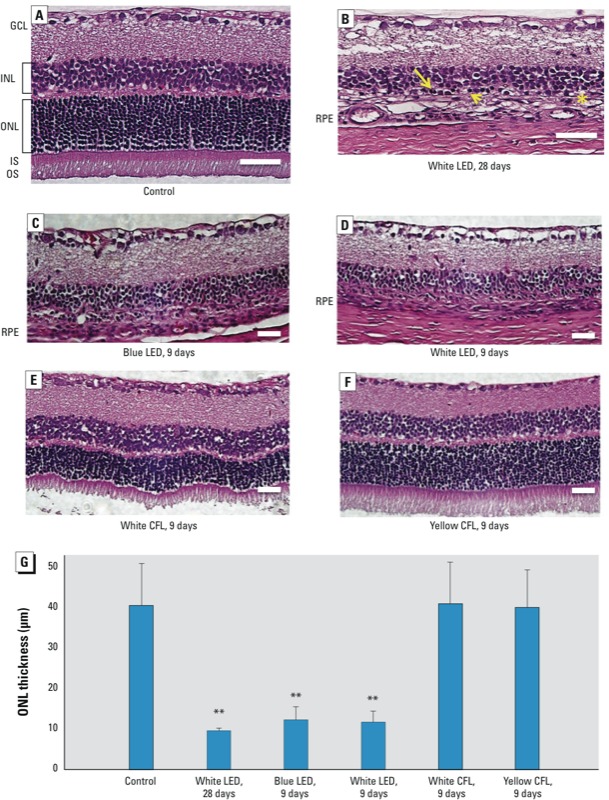
H&E staining of representative retinal tissue sections from control rats (*A*) and from rats exposed to white LED for 28 days (*B*) or to blue LED (*C*), white LED (*D*), white CFL (*E*), or yellow CFL (*F*) for 9 days. (*G*) ONL thickness (mean ± SD) measured in retinas (*n* = 3 controls, *n* = 8 for each light-exposure group at either time point). Abbreviations: GCL, ganglion cell layer; INL, inner nuclear layer; IS, inner segment; ONL, outer nuclear layer; OS, outer segment; RPE, retinal pigment epithelium (usually next to the OS layer but is detached and cannot be found within this scope). (*A*) Control tissue shows normal retinal layers. (*B*) After exposure to white LED for 28 days, retinal injury included pyknotic photo­receptor nuclei (arrow), swelling of the inner segment (arrow head), a dis­organized outer segment with no visable RPE [asterisk (*)], and INL degeneration. Photoreceptors were not present in retinals from rats exposed to blue LED (*C*) or white LED (*D*) light; the white CFL group (*E*) exhibited distortion of the OS and ONL, and the yellow CFL group (*F*) exhibited less movement in each layer. In (*A–F*), bar = 50 μm. (*G*) ONL thickness was significantly decreased in the LED groups at days 9 and 28, whereas the ONL thickness in white and yellow CFL groups was not significantly altered at day 9. **p < 0.01, compared with the control group by ANOVA and the Tukey post hoc test.

*Retinal histology.* Exposure to white LED light exposure can lead to morphologic alterations in the rat retina. Compared with the control group (Figure 4A), the white LED group exposed to 750 lux white LED light for 28 days (Figure 4B) exhibited pyknotic photoreceptor nuclei, swelling of the inner segment, and a disorganized outer segment. ONL thickness was significantly decreased at day 9 and day 28 in the white and blue LED groups (Figure 4C,D) (*p* < 0.01, by ANOVA followed by Tukey post hoc test; Figure 4G), whereas we observed no significant change in ONL thickness in the white and yellow CFL groups at day 9 (Figure 4E,F,G).

**Figure 5:**
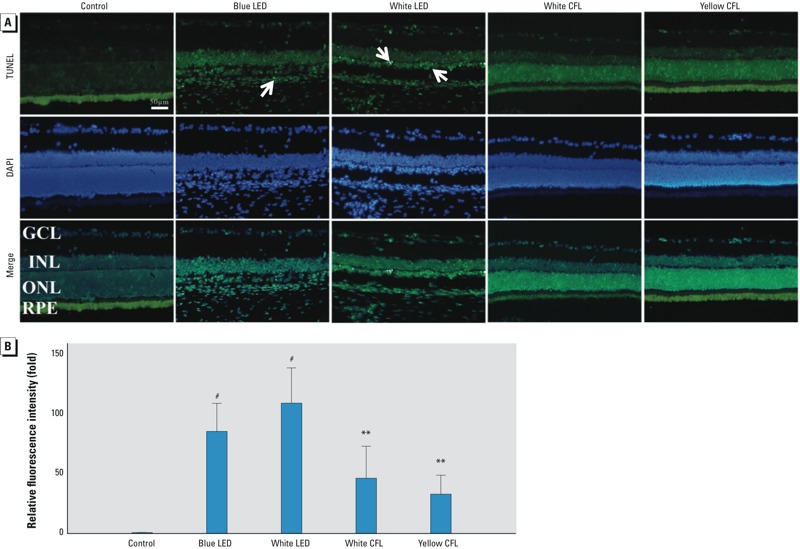
Retinal cell apoptosis detected by TUNEL labeling (damaged retinal cells show positive labeling). (*A*) Representative images of retinal cell apoptosis in control rats and in rats exposed to blue LED, white LED, white CFL, or yellow CFL for 9 days (bar = 50 μm); more apoptotic cells (arrows) appear in the retina of LED-exposed groups than that of CFL-exposed groups. Abbreviations: GCL, ganglion cell layer; INL, inner nuclear layer; ONL, outer nuclear layer; RPE, retinal pigment epithelium. (*B*) Fluorescence intensity of apoptosis in light exposure groups shown as the mean ± SD fold of the control value (*n* = 3 controls and 8 for each exposure group). The LED-exposed groups exhibit higher fluorescence intensity than that of CFL-exposed groups. **p < 0.01, and ^#^*p* < 0.001, compared with the control group by ANOVA and the Tukey post hoc test.

*Apoptosis detected by TUNEL staining.* Light exposure induced significant retinal cell apoptosis in all light-exposed groups (Figure 5A,B). However, more apoptotic cells were observed in the retina of the LED-exposed groups than in that of CFL-exposed groups after 9 days of exposure (*p* < 0.001 for LED groups and *p* < 0.01 for CFL groups, by ANOVA followed by the Tukey post hoc test) (Figure 5B).

**Figure 6:**
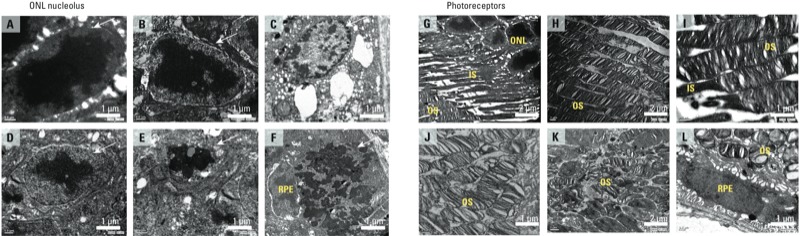
Representative TEM photomicrographs showing retinal cellular injury of the ONL nucleolus (*A–F*) and photo­receptors (*G–L*) in control rats (*A*,*G*) and those exposed to white LED light (*B–F*, *H–L*) at day 9. Abbreviations: INL, inner nuclear layer; IS, inner segment; ONL, outer nuclear layer; OS, outer segment; RPE, retinal pigment epithelium. ONL nuclear deformations (arrows) were observed in (*A*) control ONL nucleus and as (*B*) nucleolus condensation, (*C*) karyolysis, (*D*,*E*) pyknosis, and (*F*) karyorrhexis. (*G–L*) Normal photo­receptor, IS, and OS from a control rat (*G*); photo­receptor deformations showing minor disruption (*H*,*I*); and IS disappearance followed by OS shrinkage and the formation of several small round shapes (*J*,*K*,*L*). For (*A–F*) and (*I*,*J*,*L*), bar = 1 μm; for (*G*,*H*,*K*), bar = 2 μm. Each photomicrograph is from a different sample.

*TEM analysis.* Figure 6 shows nucleolar damage of photoreceptors in control tissue and in samples collected after 9 days of exposure to white LED light. Nucleolar damage of photoreceptors that occurred after exposure include an early stage of nucleolar condensation (Figure 6B), karyolysis (Figure 6C), pyknosis (Figure 6D–E), and karyorrhexis (Figure 6F). We also observed disruption of the inner and outer segments (Figure 6G–L).

**Figure 7:**
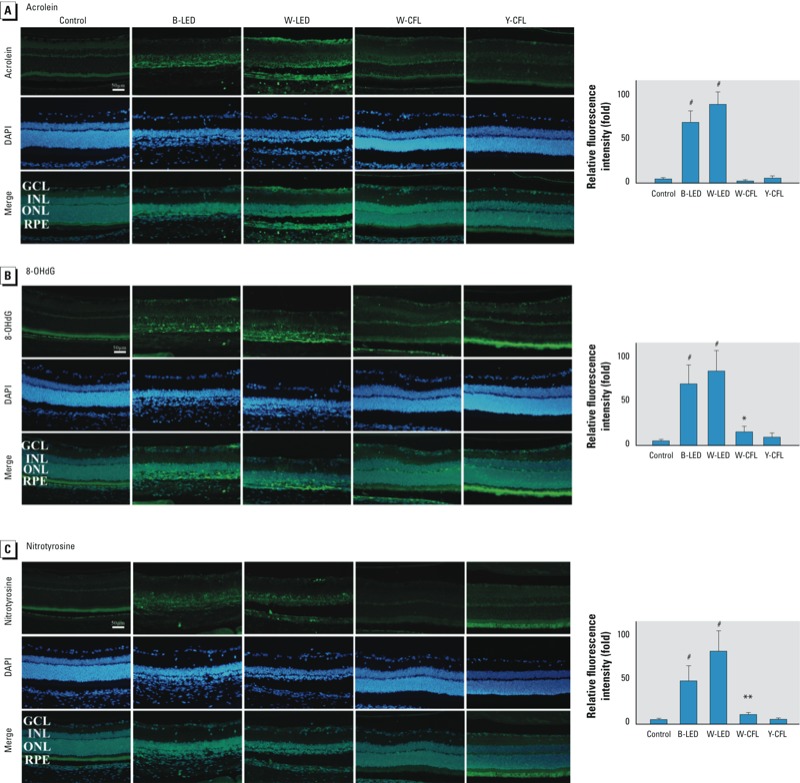
Retinal light injury shown by IHC staining for acrolein to detect lipid adducts on macro­molecules (*A*), 8-OHdG to detect DNA adducts (*B*), and nitro­tyrosine to recognize protein adducts (*C*) in the retina of unxexposed rats or rats exposed to blue LED, white LED, white CFL, or yellow CFL for 9 days. Abbreviations: B, blue; W, white; Y, yellow. Left, representative photo­micrographs (bar = 50 μm). Right, mean ± SD fluorescence of protein-positive cells relative to the control group (*n* = 3 controls, *n* = 8 for each light-exposure group). LED-exposed groups exhibited higher fluorescence intensity on ONL, and the CFL groups had lower fluorescence intensity. *p < 0.05, **p < 0.01, and ^#^*p* < 0.001, compared with the control group by ANOVA and the Tukey post hoc test.

*Immunohistochemistry.* Oxidative damage results in adducts on macromolecules that can be detected by immunohistochemistry. We used three antibodies to detect cell conditions in retinas of rats at the 9-day time point: acrolein for lipid recognition (Figure 7A), 8-OHdG for DNA detection (Figure 7B), and nitrotyrosine for protein identification (Figure 7C). LED-exposed groups exhibited higher fluorescence intensity with acrolein, 8-OHdG, and nitrotyrosine in ONL and CFL induced lower fluorescence intensity of these three proteins in ONL.

**Figure 8:**
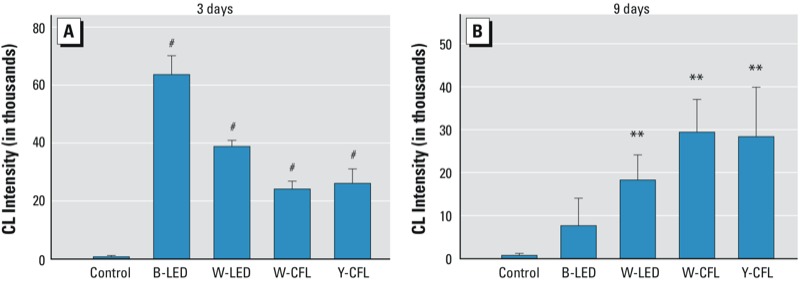
ROS assay performed in control rats and rats exposed to blue LED, white LED, white CFL, or yellow CFL for 3 days (*A*) or 9 days (*B*). Values are presented as mean ± SD chemiluminescence (CL) intensity. Abbreviations: B, blue; W, white; Y, yellow. (*A*) After 3 days of exposure to blue LED light, lucigenin-stimulated O_2_^•–^ exceeded 60,000 in total count, the white LED group had a high total count near 40,000, and the CFL groups had total counts of 20,000–30,000; At this time point, control rats exhibited a count of approximately 1,000. *n* = 3 controls, and *n* = 3 for each exposure group. (*B*) After 9 days of exposure, the O_2_^•–^ total count for the blue LED light group decreased to 8,000, that for the white LED light group decreased to 18,000, and that for both fluorescent light groups remained at the same level. *n* = 3 controls, and *n* = 8 for each exposure group. **p < 0.01, and ^#^*p* < 0.001, compared with the control group by ANOVA and the Tukey post hoc test.

*Oxidative stress.* As shown in Figure 8A, lucigenin-stimulated superoxide anion (O_2_^•–^) and total oxidative products were computed for all groups. After 3 days of exposure to blue LED light, retinal O_2_^•–^ measured 8 min after stimulation exceeded 60,000; the white-LED group exhibited a high total count close to 40,000, and the CFL groups accumulated smaller total counts, from 20,000 to 30,000. However, the plot exhibited an opposite trend when the exposure duration was increased to 9 days (Figure 8B). This result suggests that retinal oxidative stress may be induced by light exposure in the early stage of exposure.

## Discussion

Retinal light damage depends on the duration of exposure and the light level reaching the retina (retinal irradiance). The pathological process is also wavelength dependent (Organisciak and Vaughan 2010). The results of the present study indicate that exposure to LED light in this albino rat model can induce retinal damage as evidenced by the functional ERG study, IHC, TUNEL, and TEM examinations. Our results also suggest that this retinal damage could be related to blue light–induced oxidative stress within the retinal tissues, as evidenced by the ROS generated in the retina after LED light exposure.

The ERG results show functional loss in the retina after LED light exposure. The white and blue LED groups demonstrated a significant decrease in the b-wave amplitude at days 9 and 28 after light exposure. The morphological results show that exposure to cyclic white LED light may induce outer retinal damage within 9 days and may be responsible for further deterioration when the exposure duration is extended. The ONL, which is usually 12–14 rows of nuclei in unexposed Sprague-Dawley rats at 2–3 month of age, was reduced to approximately 4–5 rows. OS and IS were absent, and the RPE appeared to be damaged or missing. However, we observed less damage within the photoreceptor after exposure to yellow CFL, as shown in Figure 4F. Our functional and morphological results indicate that the wavelength and the SPD, rather than total light irradiance, are crucial risk factors that contribute to photochemical retinal injury. The results also suggest that LED light–induced cell death may occur through the intrinsic apoptotic pathway under oxidative stress. Sliney (1984) calculated that for the same lamp brightness, the retinal irradiance in the rat eye would be at least 60% greater than experienced in the human retina. The light exposure in the present study began at 1800 hours to match the nocturnal activity pattern, but this exposure time may also enhance susceptibility to light damage in rats. Therefore, the careful development of an action spectrum for LED light damage remains an important research goal.

The retina has one of the highest oxygen consumption levels of tissues in the body, and it is sensitive to oxidative stress (Yu and Cringle 2005). Oxidative stress is the crucial risk factor for photoreceptor degeneration, which is caused by the generation of toxic ROS within retinal tissue. The retina contains enzymes involved in detoxification or synthesis, particularly in the OS or RPE (Newsome et al. 1990). In the present study, we compared the phototoxicity of CFLs with that of typical white LEDs. The white LED lights carry higher energy that exceeds the threshold of the enzymes that serve as a stress-induced protection mechanism (Behar-Cohen et al. 2011); thus, exposure to these white LEDs may result in severe damage to the outer retina. To prevent or decrease this potential retinal damage, some companies are increasing the market segments of lower color-temperature LEDs for domestic lighting (U.S. Department of Energy 2012).

Photochemical damage is the major cause of low-intensity chronic exposure light-induced injury. Noell (1980) indicated that the direct action of light on photoreactive molecules within the damaged cell causes primary damage. Secondary damage, which follows the primary event, can either continue the damaging process in the same cell or expand to other cells (Noell 1980). The main concern is that light damage involves oxidative events (Lohr et al. 2006). In the present study, we used several exposure durations to analyze cause and effect in a temporal manner (Figure 1). We found that LED lights carry energy that is strong enough to generate oxidative stress (Figure 8). Our results are consistent with the observation by Noell (1980); that is, retinal neuronal cell DNA levels are correlated with ERG b-wave estimates of photoreceptor cell loss in light-exposed retinals of rats. Oxidative stress is responsible for pathogenesis of light injury, especially when light is sufficient to damage > 80% of photoreceptor cells detected by nonrecoverable ERG b-waves. Furthermore, our histological analysis showed that most cell death does not occur immediately after light exposure; the damaged retinal neuronal cells may lose function but are still present on the retinal layers with oxidative modified lipids, nucleic acids, and proteins.

## Conclusions

LEDs are expected to become the primary domestic light sources in the near future. Certain amounts of LED light exposure may induce retinal damage, and this animal model provides comparative measures of damage from different commercial light sources. Albino rats are commonly used for retinal light injury experiments (Collier et al. 2011). Retinas from rats maintained in the dark for 14 days are more susceptible to light-induced damage than normal pigmented retinas (Organisciak and Vaughan 2010). Our results show that the SPDs of bluish-white (high CCT) LEDs contain a major fraction of short-wavelength light that causes irreversible retinal neuronal cell death in rats. Furthermore, this model shows that the SPD of white LEDs now being introduced for domestic lighting pose a theoretical risk compared to CFLs (or incandescent lamps that have little blue light). When analyzing blue-light hazards, we cannot exclude the risk of chronic effects from daily exposure because photochemical damage may not induce an acute syndrome; instead, blue light exposure may cumulatively induce photoreceptor loss.

Regardless of whether the initial damage is caused by a photochemical effect, LED light damage is dependent on wavelength and duration. The entire retinal neuronal cell is affected, regardless of whether the injury is localized in the outer segment, mitochondria, or other subcellular organelles. Because illuminance levels of LED domestic light sources may induce retinal degeneration in experimental albino rats, the exact risks for the pigmented human retina require further investigation.

## Supplemental Material

(152 KB) PDF
