# Supplementary material for: White Light–Emitting Diodes (LEDs) at Domestic Lighting Levels and Retinal Injury in a Rat Model
Source: Environ Health Perspect. 2013 Dec 20;122(3):269–76. doi: 10.1289/ehp.1307294 (PMC3948037; doi:10.1289/ehp.1307294)
Supplement: (152 KB) PDF [file ehp.1307294.s001.pdf]

Environmental Health Perspectives doi:10.1289/ehp.1307294

Supplemental Material
White Light–Emitting Diodes (LEDs) at Domestic Lighting Levels and Retinal Injury in a Rat Model

Yu-Man Shang, Gen-Shuh Wang, David Sliney, Chang-Hao Yang, and Li-Ling Lee

Table of contents

Animals and rearing conditions……………………………..………………………………..2

Light source…………………….……..……..…………………….……………………………2

Light exposure……………………..……………………………………….…………...……3

Sample pretreatment……………………..…………………………………….….….…….4

Electroretinography (ERG)……..…………………………..…………………….……...…..…4

Hematoxylin and Eosin (H&E staining) ……………………...…………………...…...….…5

Terminal deoxynucleotidyl transferase dUTP nick end labeling (TUNEL)…………..……….…5

Immunohistochemistry (IHC)……..………………..………………..………..…….…..……..…6

Transmission electron microscopy (TEM) analysis…………………..………….……...…...…6

Free radical assay (reactive oxidative species)…..……….…………..………………………..…7

Statistical analysis.……….……….……………………………..………...……......……..…7

Supplemental Material, Figure S1………………………………………………………...8

Animals and rearing conditions

In total, 120 adult male Sprague–Dawley rats were purchased from BioLasco Taiwan Co., Ltd at 8 weeks of age and stored in a dark environment for 14 days to clear the light exposure effect from their previous rearing environment. Twelve normal rats served as controls without exposure, and the other 108 rats received programmed light exposure, as shown in Figure 1. The animals were randomly assigned to each of the treatment groups and were housed individually throughout the study. The room temperature was maintained at 22°C with humidity between 50% and 70%. The shelves were located in the animal laboratory of National Taiwan University School of Medicine. An isolate and locked room was set up for the experiment. Routine animal maintenance under the dark environment was conducted under dim red illumination (>600 nm) for less than 30 min/d. All animals received food and water *ad libitum*. The pelleted feed was supplied (LabDiet #5001) containing carbohydrates (53.3%), protein (23.9%), fat (10.7%), minerals and vitamins (7%), and fiber (5.1%). Fresh tap water was provided by bottle with sipper tube attached at the end. The water was analyzed for contaminations. The white alpha cellulose paper bedding (ALPHA-Dri™ #1102) was used. The use of rats in this study conformed to the ARVO statement for the Use of Animals in Ophthalmic and Vision Research and Laboratory Animal Resource Committee guidelines at National Taiwan University. The animals were treated humanely and with regard for alleviation of suffering. The averaged weights of experimental animals were recorded and are shown in Supplemental Material, Figure S1.

Light source

LED lights with varying spectral characteristics were used as the sources for primary exposure treatments; whereas, cool-white and warm-white compact fluorescent lamps (CFL) were used for comparison. As shown in Figure 2. Single-wavelength blue LEDs (460 ± 10 nm) and blue LED

with yellow phosphor-converted (PC) white LEDs were custom made for the exposure experiments. The PC-LED had a correlated color temperature (CCT) of 6500 K. One group of white CFL also matched the CCT at 6500 K (Chuanshih, ESE27D-EX, Taiwan), whereas the other group of yellow CFL was set at 3000 K (Chuanshih, ESE27L-EX, Taiwan). Each light source was programed for 40 measurements in an integrating sphere. The spectrum distributions and total intensities for all light sources were tested by the Industrial Technology Research Institute of Taiwan (https://www.itri.org.tw/eng/) a Certification Body Testing Laboratory (CBTL).

Light exposure

As shown in Figure 1, the animals were divided into 4 groups, and each rat was stored in an individual transparent cage with a dimension of 45 x 25 x 20 (cm). Each cage was placed in the center of a rack shelf with dimensions of 75 x 45 x 35 (cm). A rack with 6 shelves was covered with a black curtain to secure the lighting condition. Each group had 2 racks with dimensions of 75 x 45 x 225 (cm). The light sources were set on the top of each shelf and were measured 20 cm away from each source to acquire the luminance at 750 lux. After 14 d of dark adaptation, the light exposure started at 6:00 PM of Day 15 with the total exposure duration ranging from 3, 9, to 28 d under 12hr dark/12hr light cyclic routines. The cyclic light exposure routine set as dark during the day (6:00 AM ~ 18:00 PM) and light during the night (18:00 PM ~ 6:00 AM). The animals were sacrificed for analysis after light exposure. However, a special treatment for 32 animals was performed, 8 from each group were returned to a dark environment for 14 d of recovery after 28 d of exposure. The objective of the recovery stage was to allow for possible removal of necrotic photoreceptor cell debris. The time of measurement or sacrifice of the groups counter-balanced according to treatment.

Sample pretreatment

Animals were anesthetized, and both eyes were scanned using ERG after completing the light treatment. They were sacrificed immediately with pentobarbital sodium (>60 mg/kg, intraperitoneal) after the ERG scans. For hematoxylin and eosin (H&E staining) and terminal deoxynucleotidyl transferase dUTP nick end labeling (TUNEL) staining, enucleated eyes were immersion-fixed in 4% paraformaldehyde in 0.1 M phosphate buffered saline (PBS) at pH 7.4 overnight before paraffin-embedding. For IHC stains, eyeballs were frozen immediately inliquid nitrogenafter enucleation. Cryosections of 4 μm in thickness were made in the glass slide and maintained at -80°C until analysis. For a reactive oxygen species (ROS) assay, eyeballs were frozen immediately in liquid nitrogen after enucleation. The eyeballs were grinded with saline (500 μL saline with one eye) for extraction. Lastly, for transmission electron microscopy (TEM) analysis, the eyeballs were immersion-fixed in 2.5% glutaraldehyde in PBS for 2 hr before further processing.

Electroretinography (ERG)

Retinal electrical responses were recorded before and after light exposure using ERG (Acrivet, Hennigsdorf, Germany). After 18 hr of dark adaptation, rats were anesthetized using an intramuscular injection of 100 mg/kg ketamine and 5 mg/kg xylazine (WDT eG, Garbsen, Germany). One drop of tropicamide (0.5%) (Mydriatikum Stulln, Pharma Stulln, Germany) was applied for pupil dilation before ERG measurement. One drop of Alcaine (0.5%) (proxymetacaine hydrochloride; Alcon Pharmaceuticals Ltd, Puurs, Belgium) was applied for local anesthesia before placing the active electrode onto the cornea. Two subcutaneous needle electrodes (Ambu Neuroline Twisted Pair Subdermal, Bad Nauheim, Germany) served as the reference and ground electrodes. The reference needle was subcutaneously inserted between 2

eyes, and the ground needle was subcutaneously inserted between 2 rear legs to receive proper impedance levels, which was less than 10 kΩ at 25 Hz. LED flashes were stimulated without background illumination. Each 20-milli-second flash was provided by a 4 W LED (1 mV), and the illumination was set at 2.5 log cd·s/m^2 for Scotopic ERG response. The weighted average of 10 stimulations was computed by the program to produce the final detection values.

Hematoxylin and Eosin (H&E staining). After pretreatment, paraffin sectioning was performed after 9, 28, 28+14 days of light exposure (4% paraformaldehyde in 0.1 M phosphate buffer [pH 7.4] for 1 hr at 48°C), and the eyeballs were dehydrated in EtOH, infiltrated in Xylene, and embedded in paraffin. Radial 5 μm sections were stored at 48°C. Histologic analysis included quantification of the outer nuclear layer (ONL) and retina morphology alteration by using a light microscope. The midsuperior aspect of the retina was examined for all histological analyses in this study.

Terminal deoxynucleotidyl transferase dUTP nick end labeling (TUNEL)

The TUNEL assay was performed using a FragEL™ DNA fragmentation detection kit (Calbiochem, Darmstadt, Germany) following the standard protocol with a minor modification to detect apoptotic cells after 9 d of light exposure. Tissue sections were deparaffinized, rehydrated, and blocked using endogenous peroxidase with H_2O_2 for 30 min. Antigen retrieval was achieved by pressure-cooking in a 0.1 M citrate buffer at pH 6 for 10 min followed by cooling at room temperature before incubation with the enzyme. The TUNEL enzyme (1 hr at 37°C) and peroxidase converter (30 min at 37°C) were applied to the 10-μm sections after incubation in a permeabilizing solution of 0.1% Triton-X in 0.1% sodium citrate for 5 min. The tissues were counter-stained with DAPI, and the fluorescent signals were obtained by adding FITC-Avidin,

which bound to the biotinylated-dU of the damaged DNA. Sections were visualized on a fluorescent microscope over the entire retina excluding the RPE layer (Nikon Instruments Inc., NY, USA). The number of TUNEL-positive cells for each section was counted by Image-Pro Plus software (v.6.0). The number of TUNEL-stained nuclei quantified in 4 random slides per sample.

Immunohistochemistry (IHC)

Cryosections of the retina samples were incubated overnight at 48°C with specific primary antibodies. Three antibodies were used for detection as follows: 8-hydroxy-2'-deoxyguanosine (8-OHdG) (1:50, JAICA, Japan) for DNA, acrolein (1:200, Advanced Targeting Systems, San Diego CA USA) for lipids, and nitrotyrosine (1:200, Abcam, Millipore Billerica, MA USA) for proteins. The number of IHC-positive cells for each section was counted by Image-Pro Plus software (v.6.0).

Transmission electron microscopy (TEM) analysis

TEM was performed at the Electron Microscopy Facility at the Department of Pathology at National Taiwan University Hospital (Taipei, Taiwan). Retina slices at 1 mm were prefixed in 2.5% glutaraldehyde in PBS, postfixed with 2% osmium tetroxide, and dehydrated for 10 min each in sequential baths of 30%, 50%, 70%, 90%, and 100% ethanol. The specimens were placed into propylene oxide for 30 min, followed by a mixture of propylene oxide and epoxy resin for an additional 1 hr; the samples were subsequently embedded into a gelatin capsule with epoxy resin at 60°C for 1 d. Subsequently, 80 to 90 nm ultrathin sections were obtained using ultramicrotome. The sections were stained with 2% tannic acid in distilled water (DW) for 5 min followed by 2% uranyl acetate in DW for 15 min and a lead-staining solution for 5 min. In the final step, sections were coated with thin copper grid-film and placed in a vacuum chamber for

scanning. The specimens were examined using TEM with a high-resolution instrument at 80 kV (JEOL JEM-1400, Japan).

Free radical assay (reactive oxidative species)

We loaded 0.2 mL of homogenized extraction with 0.1 mL of 0.9% saline onto a 3 cm dish with a stirring bar placed at the center. The dish was placed into the chemiluminescence analyzer chamber (Tohoku CLA-FS1, Japan). reactive oxygen species were quantified after adding the enhancers lucigenin (bis-N-methylacridiniumnitrate) to the chemiluminescence analyzer (Tohoku CLA-FS1, Japan). After 1 min of background detection, 1 mL of a lucigenin solvent (2.5 mg of lucigenin dissolved in 50 mL 0.9% saline) was added for stimulation. The stimulated superoxide anion (O_2^-) and total oxidative products were captured every 10 s and computed for 7 min.

Statistical analysis

Data were presented as the mean ± SD unless otherwise stated. Data were evaluated using analysis of variance (ANOVA) with Tukey post hoc tests to show differences between the groups. A p value less than 0.05 was considered to be statistically significant.

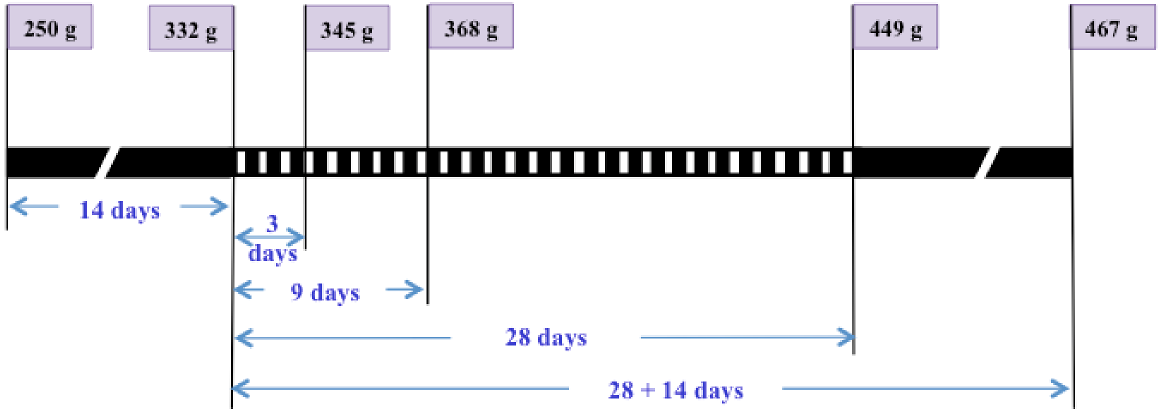

Supplemental Material, Figure S1. Average weights of experimental animals.
